#  Work Predictors of Lapse in Patients under Treatment of Methadone Maintenance Therapy

**Published:** 2015-09

**Authors:** Ramin Mehrdad, Benafsheh Zarbafi, Gholamreza Pouryaghoub, Maryam Saraeei

**Affiliations:** 1 Center for Research on Occupational Diseases, Tehran University of Medical Sciences, Tehran, Iran; 2 Occupational Medicine Resident, Tehran University of Medical Sciences, Tehran, Iran

**Keywords:** *Methadone Maintenance Therapy*, *Lapse*, *work*, *Predictors*

## Abstract

**Background:** Addiction to narcotics ‎can cause serious problems and ‎monetary losses. Therapeutic ‎success can be predicted ‎through identifying lapse risk ‎factors.‎

**Objective:** Determining Job Risk ‎Factor of Lapse.‎

**Methods: **This was a cross ‎sectional study on 351 addicts ‎visiting five methadone clinics. A ‎Data collection sheet consisting ‎of questions on demographic ‎and job information was filled up ‎through direct interviews. ‎Information relating to lapse in ‎the first month was analyzed.‎

**Results:** The mean (SD) age of ‎the participants was 40(12) ‎years; of them, 74% were ‎employed, of whom 34% had ‎lapsed. A relationship was ‎noticed between education ‎qualification (secondary school ‎compared with higher and lower ‎education) and lapse (p = .04), ‎and also between type of drug ‎abuse (amphetamine) and lapse ‎‎(p = .001).‎

**Conclusion:** **‎** **‏** ‏‎ Lapse was more ‎dependent on type of drug abused, ‎and employment had a protective role, ‎with no correlation with the type of ‎job and other job conditions. Non-‎work factors mediated/suppressed the ‎role of occupational conditions.‎

Drug addiction is common and can cause serious monetary losses due to irregular attendance at work, poor work quality, tension with colleagues and ignoring the safety measures that can cause occupational accidents (1). By identifying factors such as personal character, job situation, family and personal situation of the individual, lapse to addiction can be predictable; and therefore, it may be prevented by taking appropriate and timely measures (2). 

Substance abuse increased in workplace (from7% to 20%) (19). the population of Iran at present is 75 million, of whom; 24 million are employees (12). According to optimistic estimates, 10 % of these people are gripped with the problem of addiction (3). Various factors are responsible for the spread of addiction and abuse of drugs, and the interaction of these factors causes initiation and then the addiction to drugs (4). Treatment with medication is the first choice adopted by Iranian physicians. One such treatment method is the use of methadone as a substitute which takes several months.

Job factors that can lead to lapse during treatment period are as follows: Heavy work, shift working, the job contract, lack of insurance benefits (5), being away from family, easy access to drugs, tough climatic conditions, lack of a strict policy about drug consumption (6), job security, job rigors (7), type of job, and long working hours(8). In general, factors responsible for the relapse of addiction vary to some extent in different studies which can be due to the influence of socio-economic and cultural conditions prevailing in the society under study. Therefore, separate studies should be conducted in every society to determine addiction factors. Because of the significant differences in industrial jobs, such a study is particularly more necessary. Moreover, studies undertaken on job factors have been performed on addicted persons without taken into account their discontinuation of addiction, i.e., some studies have been done in such a manner that the factors leading to the initiation or relapse of addiction in the workplace was measured (factors outlined above) whereas very few studies exist on the relapse during treatment which is termed as 'lapse'. In fact, this study aimed to identify these factors during treatment, and to find an answer to this question that can a person who is surrounded by mentioned risk factors, be treated successfully or should he/she change their job. 

Many patients who are under treatment with methadone, which may take several years are employed, so while at work, they should take daily doses of this medication. Furthermore, their work environment should be suitable and help them to continue and succeed in their treatment. Most clinicians believe that these patients should do light jobs during the first month of the treatment; and if possible, should not work during this period. If the patient is jobless, he should busy himself with some job during the second month of the treatment. There are no references to this, but many therapists suggest it based on their experience. Hence, the present study was conducted to identify those job factors that may cause lapse during the first month of the treatment and also, to find whether this recommendation is reasonable or not.

## Materials and Method

This was a cross sectional study conducted on 351 male addicts visiting the methadone treatment centers having already passed one month of the treatment. 

The participants were selected from five methadone treatment centers in Tehran province, whose staff willingly cooperated with us after being briefed about the study. From 100 methadone clinics in Tehran, 5 clinics were randomly selected. The participants were from among the visitors of the morning as well as the evening shift and their visit frequency varied from daily to weekly visits depending on their month of treatment. Therefore, it was necessary that this data collection be carried out through a period of six months (October- March, 2012). Considering the sensitiveness and privacy of the matter, only patients willing to participate were selected for the study, and a data collection sheet(was performed by reviewing the article) was filled up for them by a psychologist or the physician of the center in a direct interview. Women were excluded. The data sheet had four sections: The first section consisted of demographic information such as age, marital status, familial support, and substance abuse in the first degree relatives; the second section consisted of information related to addiction of the participant such as the type of drug, age of beginning the use of substance, period under treatment, the time of receiving methadone; the third section consisted of information related to the job of the participant such as type of job, incom level, employment period, and working conditions which might have led to relapse such as shift working, job stress, working far from family, working hours, substance abuse with coworkers, existence of fun or sport in workplace, working alone; and finally questions about other diseases (psychological disorders, low back pain & other musculoskeletal disease and sexual problems) of the participant. In order to ensure the ethical conduct of the research, all cases were studied and analyzed under code numbers. Lapse cases were recorded following confession by the participant or after an addiction test result. The participants were allowed to decline answering any question they were not comfortable with; hence the term 'missing' is frequently seen in the data sheet for some of the questions, which was inevitable considering the nature of the study. After the data sheets were completed, data were analyzed using SPSS 11.5 program, and linear regression analysis model was also utilized. Analysis of lapses during the first month of treatment was performed. During the first month, some of the participants were employed and some were jobless; and working conditions of the employed participants were investigated. Our protocol was approved by the Ethical Committee of Tehran University of Medical Sciences.

## Results

The total number of persons studied was 351 with mean (SD) age of 40 (12) years, with a mode of 43 years. The mean (SD) age of beginning the use of substance was 24 (8) years with a mode of 18 years. The minimum age of initiation was 12 years, and the maximum was 67 years. The mean and standard deviation of employed age was 21 and 12 years, respectively and the maximum age frequency was 20 years. 

With respect to the month of treatment, the latest of them was in the first month of treatment (9.1%) and the earliest of them was in the 84th month of treatment (0.6%). Most of the participants were in the first six month period of their treatment. 

Table 1 demonstrates the qualitative variables in this study. Examination of diseases had the following results: Of the participants, 23.9% suffered from back pain; 20.1% had other musculoskeletal problems such as joint and muscle pains; 26.4% revealed sexual problems, the details of which were not enquired; 47.3% had sleep problems, out of which 84.6% could not go to sleep and suffered from shortage of sleep; 4.7% slept more than normal and 10.7% had other problems such as apnea, nightmares, and lack of proper continuous sleep; 52.4% suffered from sleep problems even before the start of treatment; and from all those who had sleep problems, only 19% took medication. 

 The average number of off days was about one day, most of which was on Friday. Least off-duty persons were those who worked without holidays such as salesmen, and the most off-duty persons were those who worked on part-time jobs. 

Only 15.7% of the participants were conscious about the consequences of using narcotics. Moreover, 66% of the employed participants believed that precautionary measures at work-place are helpful; and 42.1% agreed to take an addiction test at their work-place. The main jobs of the participants under study were as follows: Driving 14.7%, construction related jobs such as masonry, wiring, etc 10.1%, salesmen 14.3%, worker 12.2%, and etc.

With respect to working hours, the minimum working hours were 3hrs/day, and the maximum working hours were 24 hrs which belonged to janitors. Average working hours were 10.46 hours, and most of the participants worked for 8 hours.

**Table 1:** Frequency of the Variables that may lead to Lapse on practitioner

**Variable **	**Answer**			
	**Yes**		**No**	
Drug testing at workplace	% 18.1	N 47	% 81.9	N 213
Drug abuse by coworkers	39.6	103	60.4	157
Shift work	13	34	87	226
Working with Job stress	49.6	128	50.4	132
Speaking with employer about work problem	42	84	58	116
Work alone	30.5	79	69.5	180
Employer fired the worker( if found out about the addiction of the worker)	56.4	110	43.6	85
Have a work problem	31.3	79	68.9	175
Work ambiguity	32.3	83	67.7	174
Availability of substance in workplace	35	73	65	179
Working far from family	20.3	53	79.7	207

**Table 2:** Demographic Information of the Participant who under treatment of methadone maintenance therapy

**Variable**		**N**	**%**
Marriage Status			
	Single	91	25.9
	Married	260	74.1
Education			
	Primary school	151	43.0
	Secondary school	155	44.2
	Diploma & more	45	12.8
Type of drug abuse			
	Opium	173	62.9
	Heroin and combination	53	19.3
	Amphetamine	49	17.8
Employment status			
	Employed	260	74.1
	Unemployed	91	25.9
Type of job			
	Driving	42	14.6
	Physical works	122	42.7
	Sedentary works	122	42.7

**Table 3:** Linear Regression Analyses Model for Variables that effect Lapse in the First Month of the Treatment

	**Sig.**	**S.E.**	**Exp(B)**
Opium abuse	.269	.814	.407
Have sexual dysfunction/don’t have	.628	.564	.761
Necessity of preventive program at workplace	.482	.623	1.549
Employment status/unemployed	.353	.641	1.814
Being away from family	.049	.836	5.187
Have sleep problems/don’t have	.039	.536	.330
Knowledge about addiction	.905	.729	.916
age	.961	.027	.999

R² = 0.24

Job satisfaction level which was judged by marks from 1 to 10, had an average mark of 7.22; and of the participants, 8.7% had a job accident before starting treatment compared with 7.5% who had a job accident after starting treatment.In the first month, 34% of the participants had lapses, the maximum of which was for 30 times. The average number of lapses in the first month was about one time. 

In this study, a significant relationship was found between employment and lapses in the first month of the treatment (p = 0.036), which means the participants who were employed had lesser lapses. Moreover, type of drug used also had a significant role. The participants who used crystal meth or methamphetamine, had the maximum lapses (p = 0.001). Also, education levels had a role such that those participants having high and middle school education had higher lapses than those having primary education (p = 0.045). In the regression analysis model (Table 2), it was found that to predict lapses based on certain independent variables in the employed participants, only the type of drug had an influence on lapses (R = 0.243).

## Discussion

There are about 282 methadone clinics (Except 126 in prisons) and 38104 patients under treatment of methadone maintenance therapy in Iran, and there is an increasing trend in the number of methadone clinics nationwide. Currently, about 6000 physicians are trained and working in methadone clinics. According to this study, lapse in the first month was 34%, which is not far from what the other studies have revealed. For example, in the Siraj et al. study, relapse was reported to be 48.9% (9); and in Taqwa et al. study, it was reported to be 18.5% (9). Of course, with an increase in the use of stimulators (10), relapse rate is expected to rise higher than the present level. In addition to this, 25.9% of the patients were jobless, compared to the study conducted by Moghadam 

(59.5%) (20); and this may be due to the fact that that study have been performed on all patients, whereas ours only included those who had accepted to abandon addiction and bear the cost of their treatment. In his study, it was found that persons who had to remain awake for longer hours and spent more physical energy were more inflicted with addiction, whereas in our study such a relationship was not observed.

In the study conducted by Fallahzadeh et al., it was reported that one of the most important measures for the prevention of addiction is the provision of jobs, from the outlook of the addicts. However, in their study, no significant relationship was found between the relapse of addiction and employment (11), whereas in ours and also in many other similar studies, employment alone significantly reduced relapse (p = 0.02). In a cohort study conducted by Karen et al. on addicted doctors, similar to our own study, age did not have any influence on relapse, but a positive family history and also the use of opium along with specific psychological disorders were very important in relapse to addiction (14). It was witnessed that the use of opioids alone, as witnessed also in our study, was not the cause of an increase in relapse. In the same way, the type of expertise and performance of the participant did not have any influence on relapse.

According to Krik J study, non-work factors mediated/suppressed the role of occupation and work organization conditions. In addition to psychosocial factors, sleep disturbance is a universal risk factor for relapse (21).

## Limitations

Due to the confidentiality of addiction field, many limitations may have existed. For example, in order to fill out the data collection sheets, only few MMT centers cooperated with us. Disaffiliation to explain the working conditions was the most limitation. Despite the extensive literature review on job predictors of lapse in patients under treatment of addiction, there are still many unknown confounding factors which lead to the lack of significant results.

## Conclusion

In spite of all kinds of investigations conducted on factors causing the risk of addiction or its relapse, concluding that only 24% of the factors are predictable, does not seem to be strange as addiction is a multi-factorial disease and its main cause is psychological disorders. Moreover, according to the study conducted by Mc Lenal et al., success of treatment dependon a great extent to treatment services, and medication alone may not be very effective (13).
